# Profibrogenic Gremlin-1 expression in prostate cancer and the clinicopathologic association

**DOI:** 10.3389/fonc.2026.1910229

**Published:** 2026-08-21

**Authors:** Danqing Ren, Yu-Hsuan Chien, Reina Xu, Kai-Yuan Tsung, Gustavo E. Ayala, Tien C. Ko, Yanna Cao, Run Wang, Tung Shu

**Affiliations:** 1 Division of Urology, McGovern Medical School, The University of Texas Health Science Center at Houston, Houston, TX, United States; 2 Division of Urology, Department of Surgery, Linkou Chang Gung Memorial Hospital, Taoyuan, Taiwan; 3 Washington University in St. Louis, St. Louis, MO, United States; 4 Rice University, Houston, TX, United States; 5 Department of Pathology and Laboratory Medicine, McGovern Medical School, The University of Texas Health Science Center at Houston, Houston, TX, United States; 6 Department of Surgery, McGovern Medical School, The University of Texas Health Science Center at Houston, Houston, TX, United States; 7 Department of Urology, University of Texas MD Anderson Cancer Center, Houston, TX, United States; 8 Vanguard Urologic Institute, Houston, TX, United States

**Keywords:** epithelial-stromal interaction, extracellular matrix, grem1, prostate cancer, TCGA, tissue microarray, tumor microenvironment

## Abstract

**Background:**

Gremlin-1 (GREM1) is a profibrogenic molecule involved in TGF-β signaling. Recent studies have implicated GREM1 in androgen receptor (AR)-independent signaling and castration resistance in advanced prostate cancer. However, its compartment expression patterns and clinicopathologic significance in primary prostate cancer remain unclear.

**Methods:**

GREM1 mRNA expression and clinicopathologic associations were analyzed in the Cancer Genome Atlas (TCGA) prostate adenocarcinoma (TCGA-PRAD), the Memorial Sloan Kettering Cancer Center (MSKCC), and the German Cancer Research Center (DKFZ) primary prostate cancer cohorts. Correlations between GREM1 and genes related to TGF-β signaling, extracellular matrix organization, fibroblast activation, and AR signaling were evaluated by Spearman analysis. GREM1 protein expression was examined by immunohistochemistry in commercial human prostate cancer tissue microarrays (TMAs) using compartment-specific QuPath-based H-scores.

**Results:**

GREM1 expression was relatively elevated in prostate and bladder cancers. Across the three prostate cancer cohorts, higher GREM1 expression was associated with adverse pathologic features and was most consistently correlated with FAP. Inverse correlations were observed with selected AR-related genes, whereas no significant correlation was found with AR itself. Higher GREM1 expression was associated with shorter disease-free survival only in MSKCC but was not an independent prognostic factor after clinicopathologic adjustment. Quantitative immunohistochemistry in 43 patients showed higher epithelial than stromal GREM1 H-scores (median, 3.10 vs 1.41; P < 0.0001), with heterogeneous staining in both compartments. Neither epithelial nor stromal H-scores were associated with Gleason score or pathologic T stage.

**Conclusions:**

GREM1 mRNA expression in primary prostate cancer was associated with adverse clinicopathologic features, and a fibroblast-associated transcriptional context but did not demonstrate independent prognostic value. At the protein-level, GREM1 expression was heterogeneous in both epithelial and stromal compartments, with higher epithelial H-scores on average. These findings support further investigation of the biological significance of GREM1 expression in primary prostate cancer.

## 1 Introduction

Prostate cancer is one of the most commonly diagnosed malignancies in men worldwide and is characterized by marked heterogeneity in clinical behavior, ranging from indolent disease to aggressive, lethal phenotypes (1, 2). Although many patients with localized tumors achieve durable control with surgery or radiation, a substantial subset eventually develop recurrent or progressive disease. Progression to castration-resistant prostate cancer (CRPC), including androgen receptor (AR)-independent states driven by lineage plasticity, remains a major cause of prostate cancer mortality (3, 4). Beyond tumor-intrinsic genetic alterations, increasing evidence suggests that signals from the tumor microenvironment also contribute to disease progression and therapeutic resistance.

Gremlin-1 (GREM1) is a profibrogenic molecule involved in TGF-β/SMAD signaling (5), extracellular matrix remodeling, and tumor progression. It has emerged as a biologically relevant mediator in several solid malignancies. In prior studies, GREM1 has most commonly been described as a stromal factor expressed by cancer-associated fibroblasts (5–8), where it is linked to extracellular matrix remodeling and tumor-promoting paracrine signaling. Notably, GREM1 has also been detected in tumor cells across multiple cancer types, including glioblastoma and hepatocellular carcinoma (9), and in both stromal and epithelial compartments in pancreatic ductal adenocarcinoma (5), indicating that its tissue distribution is not restricted to stromal compartments. In prostate cancer, recent work has implicated GREM1 in lineage plasticity and castration resistance through FGFR1-related signaling under conditions of reduced AR activity (10). Spatial studies have also suggested that GREM1 may be enriched in fibrotic tumor microenvironment of prostate cancer (11).

However, despite these mechanistic insights, the clinicopathologic significance and compartment-specific expression of GREM1 in human prostate cancer remains incompletely defined. In particular, its relationship with clinicopathologic features, clinical outcomes, and AR-associated transcriptional programs in human primary prostate cancer has not been systematically defined. To further investigate these questions, we integrated transcriptomic analyses from the Cancer Genome Atlas (TCGA) prostate adenocarcinoma (TCGA-PRAD) and two independent primary prostate cancer cohorts, the Memorial Sloan Kettering Cancer Center (MSKCC) and the German Cancer Research Center (DKFZ), with protein-level evaluation in prostate cancer tissue microarrays (TMAs). We examined whether GREM1 expression is associated with Gleason score and pathologic T stage, and clinical outcomes, assessed its correlations with TGF-β signaling genes, fibroblast/extracellular matrix markers, and canonical AR-related genes, and quantified its expression separately in epithelial and stromal compartments of tumor tissues.

## 2 Materials and methods

### 2.1 Public transcriptomic cohorts

Transcriptomic and corresponding clinicopathologic data were obtained from cBioPortal (https://www.cbioportal.org/). The TCGA-PRAD PanCancer Atlas and Firehose Legacy cohorts (accessed September 25, 2025) were used for the primary analyses, depending on the availability of specific clinicopathologic variables. GREM1 mRNA expressions, reported as RSEM (batch-normalized from Illumina HiSeq RNA-SeqV2), were log2(RSEM + 1)-transformed prior to analysis. Two additional independent public cohorts (accessed July 1, 2026), MSKCC (12) and DKFZ (13), were used for external validation. The MSKCC cohort included Affymetrix Human Exon 1.0 ST microarray mRNA expression data, disease-free survival data and clinicopathologic variables. Only primary cases were used, and the metastatic cases were excluded. GREM1 mRNA expressions were reported as log2-transformed whole-transcript mRNA expression values (Affymetrix Human Exon 1.0 ST array). The DKFZ cohort included RNA-seq RPKM expression data together with biochemical recurrence-free survival and clinicopathologic data. GREM1 mRNA expressions, reported as RPKM values, were log2(RPKM + 1)-transformed prior to analysis.

### 2.2 Cross-tumor comparison of GREM1 mRNA expression

To compare GREM1 mRNA expression across genitourinary malignancies, the TCGA-PRAD PanCancer Atlas cohort was used to analyze bladder cancer (BLCA, n = 299), prostate cancer (PRAD, n = 493), testicular germ cell tumor (TGCT, n = 149), and kidney renal clear cell carcinoma (KIRC, n = 324). For BLCA and KIRC, only male samples were included to ensure sex consistency across tumor types. Differences among tumor types were assessed using the Kruskal–Wallis test followed by Dunn’s multiple comparisons test.

### 2.3 Clinicopathologic association analysis

The association between GREM1 expression and clinicopathologic variables (Gleason score and pathologic T stage) was examined in three independent cohorts: TCGA-PRAD (Firehose Legacy), MSKCC, and DKFZ. Tumors were categorized as lower/intermediate grade (Gleason score < 8, n = 292) or high grade (Gleason score ≥ 8, n = 205). For pathologic stage analyses, tumors were grouped as organ-confined disease (T2, n = 188) or locally advanced disease (T3-T4, n = 303). Comparisons between the two groups were performed using the Mann-Whitney U test. Normality was assessed using four tests (D’Agostino-Pearson, Anderson-Darling, Shapiro-Wilk, and Kolmogorov-Smirnov) (**Supplementary Table 1**). All groups in the TCGA-PRAD cohort deviated significantly from a normal distribution (P < 0.05). In the smaller MSKCC and DKFZ cohorts, normality results varied across tests and clinicopathologic subgroups. Because normality could not be established consistently after transformation, the Mann–Whitney U test was used for all three cohorts. All available cases with Gleason score or pathologic T stage data were included in these analyses. A two-sided *P* < 0.05 was considered statistically significant.

### 2.4 Gene expression correlation analysis

Spearman correlation analysis was performed between GREM1 expression and 13 genes, including TGF-β signaling components (TGFB1, TGFB2, TGFBR1, TGFBR2, SMAD2, and SMAD3), fibroblast/ECM markers (FAP and ACTA2), and androgen receptor (AR) -related genes (AR, KLK3, KLK2, TMPRSS2, and NKX3-1). Analyses were conducted in the TCGA-PRAD (Firehose Legacy, n = 493), MSKCC (n = 131), and DKFZ (n = 82) cohorts. Within each cohort, P values from the 13 correlation tests were adjusted using the Benjamini-Hochberg false discovery rate method. A two-sided adjusted q value < 0.05 was considered statistically significant.

### 2.5 Survival analysis and multivariable Cox regression

Survival analyses were performed using the endpoint available for each cohort: progression-free survival (PFS) for TCGA-PRAD (*n* = 493), following the recommendation of the TCGA Clinical Data Resource (14); disease-free survival (DFS) for the MSKCC cohort (*n* = 131); and biochemical recurrence(BCR)-free survival for the DKFZ cohort (*n* = 82). For Kaplan-Meier analysis, patients were divided into GREM1-high and GREM1-low groups using the median expression value, and survival curves were compared using the log-rank test.

Univariable and multivariable COX proportional hazards regression analysis were performed with GREM1 expression analyzed as a continuous variable. The multivariable models were adjusted for Gleason score (<8 vs. ≥8) and pathologic T stage (T2 vs. T3-T4), which were the clinicopathologic variables consistently available across the three cohorts. Hazard ratios (HRs), 95% confidence intervals (CIs), and two-sided P values were reported.

### 2.6 TMA immunohistochemistry

Immunohistochemistry for GREM1, prostate-specific antigen (PSA), and α-smooth muscle actin (α-SMA) was performed on three commercially available consecutive human prostate cancer TMAs (Cat# PRO091-01A, US BIOLAB, Germantown, MD, USA), comprising a total of 91 tumor cores from 46 adenocarcinoma cases and one matched normal adjacent tissue (NAT). A custom-made human GREM1 antibody developed in our laboratory was used for immunohistochemistry. Antibody specificity was validated previously by Western blot, which showed a single 22-kDa band, and by GREM1 siRNA knockdown, which reduced the signal by approximately 90% (5). PSA and α-SMA served as epithelial and stromal reference markers, respectively, to facilitate compartmental identification. Cores with inadequate tissue preservation or technically unsatisfactory staining were excluded. After quality control, 87 tumor cores were included in the final analysis.

GREM1 immunoreactivity was quantified separately in epithelial and stromal regions using QuPath digital image analysis (version 0.7.0) (15). Epithelial and stromal regions of interest were manually annotated, and automated positive-cell detection was performed using consistent detection parameters across all evaluable cores. Staining intensity was classified as weak (1+), moderate (2+), or strong (3+), and an H-score was calculated for each compartment as follows: H-score = 1 × percentage of weakly stained cells + 2 × percentage of moderately stained cells + 3 × percentage of strongly stained cells, yielding a continuous score from 0 to 300. Regions of interest were reviewed to exclude non-tumor tissue, artifacts, and technically inadequate areas. For patients represented by multiple evaluable cores, epithelial and stromal H-scores were averaged separately to obtain one patient-level value for each compartment. After aggregation, 43 patients had paired epithelial and stromal measurements. Compartment assignment and image-analysis results were reviewed by two investigators (YHC and DR) blinded to the clinicopathologic data, with discrepant cases resolved by consensus with a third investigator (YC). Patient-level epithelial and stromal H-scores were treated as continuous variables. Paired epithelial and stromal H-scores from the same patients were compared using the Wilcoxon matched-pairs signed-rank test. Associations of compartment-specific H-scores with Gleason score (<8 vs. ≥8) and pathologic T stage (T2 vs. T3-T4) were evaluated using the Mann-Whitney U test.

### 2.7 Statistical analysis

Statistical tests specific to each analysis are described in the corresponding sections above. All analyses were performed using GraphPad Prism version 10 (GraphPad Software, San Diego, CA, USA). All tests were two-sided, and P < 0.05 (or, where multiple-testing correction was applied, q < 0.05) was considered statistically significant.

## 3 Results

### 3.1 GREM1 mRNA expression is elevated in primary prostate cancer and bladder cancer relative to other genitourinary cancer types

**Figure 1:**
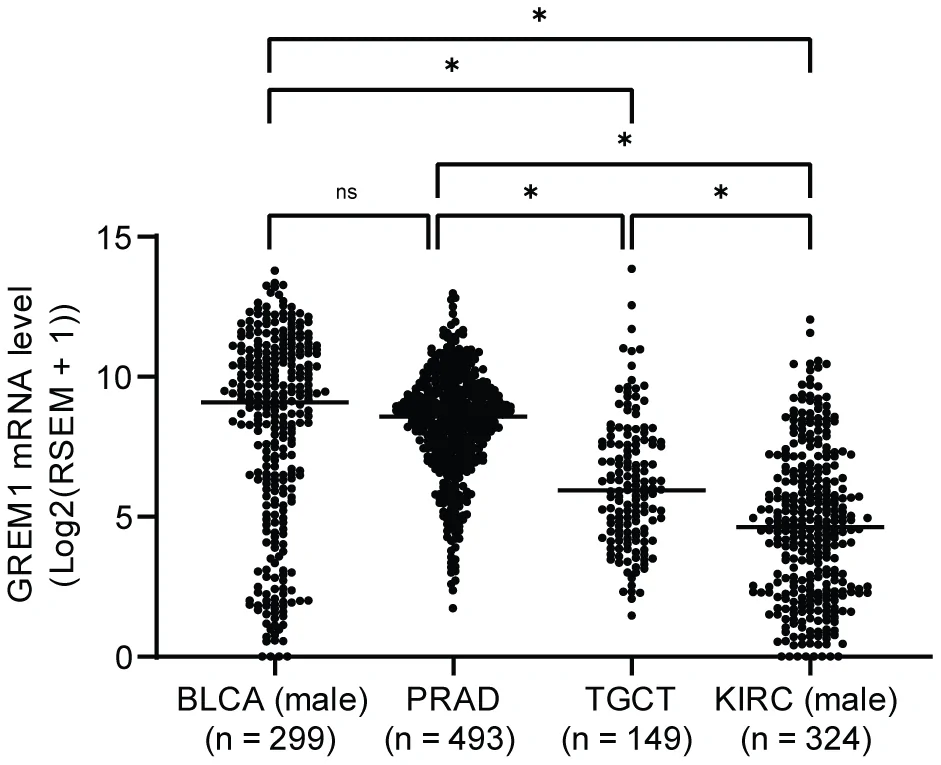
GREM1 mRNA expression across four genitourinary cancer types. GREM1 expression was analyzed in bladder cancer (BLCA, n = 299), prostate cancer (PRAD, n = 493), testicular germ cell tumors (TGCT, n = 149), and kidney renal clear cell carcinoma (KIRC, n = 324). For BLCA and KIRC, only male samples were included. Data were derived from the Cancer Genome Atlas (TCGA) PanCancer Atlas cohort. Data are shown as individual samples with median values. Statistical analysis was performed using the Kruskal–Wallis test followed by Dunn’s multiple comparisons test. *P < 0.05; ns, not significant.

GREM1 mRNA expression was first evaluated across several genitourinary cancer types using the TCGA PanCancer Atlas cohort. As shown in **Figure 1**, GREM1 expression levels were comparable between prostate cancer and bladder cancer (P > 0.05). In contrast, both prostate cancer and bladder cancer exhibited significantly higher GREM1 levels compared to TGCT and KIRC (P < 0.05). These findings indicate that GREM1 mRNA expression is relatively elevated in prostate cancer and bladder cancer compared with TGCT and KIRC.

### 3.2 GREM1 mRNA expression is associated with adverse clinicopathologic features in primary prostate cancer across independent cohorts

**Figure 2:**
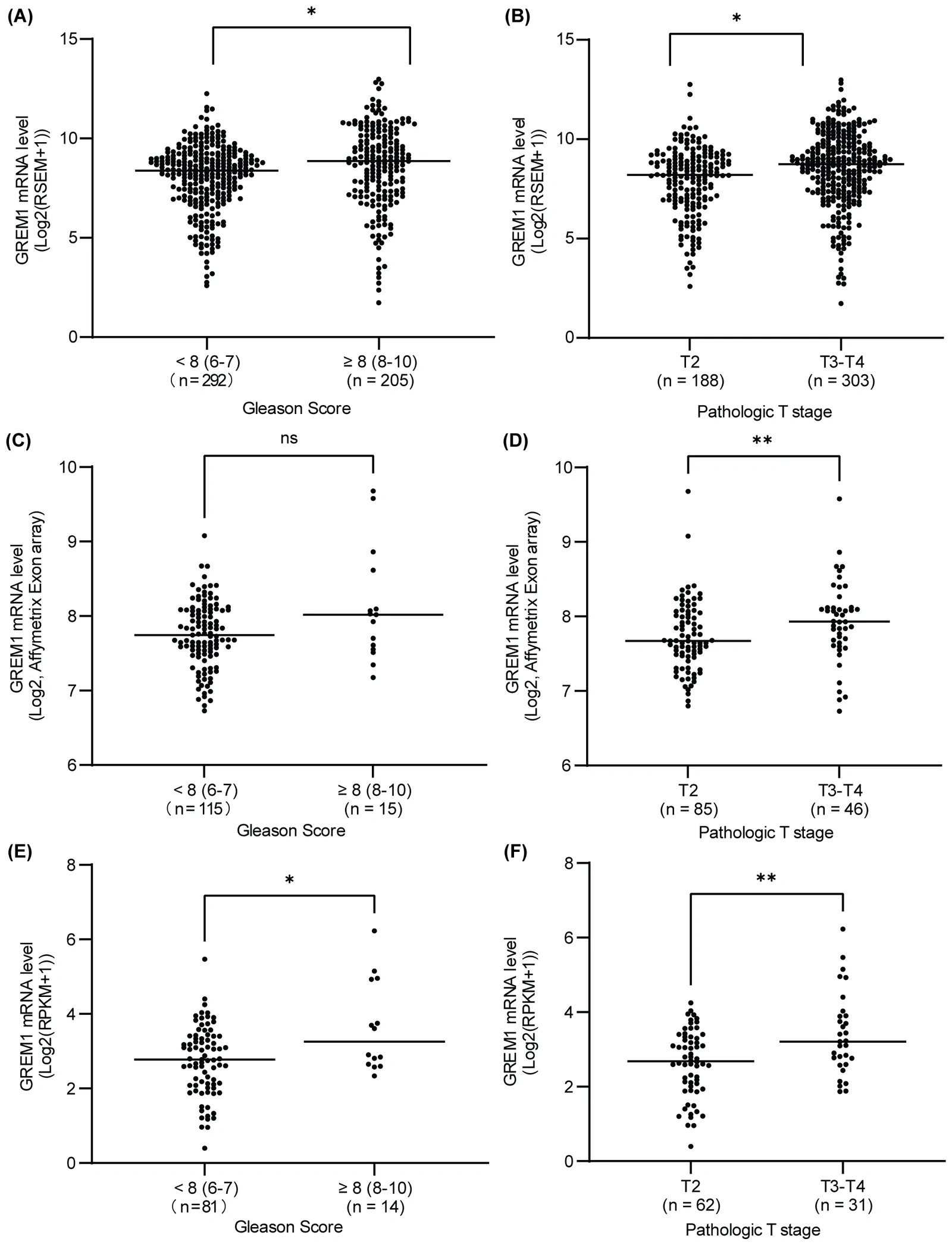
Association of GREM1 mRNA expression with Gleason score and pathologic T stage in prostate cancer. **(A, B)** TCGA-PRAD (Firehose Legacy): GREM1 expression with Gleason score and pathologic T stage. **(C, D)** MSKCC: GREM1 expression with Gleason score and pathologic T stage. **(E, F)** DKFZ: GREM1 expression with Gleason score and pathologic T stage. Data was shown as individual samples with the median. Comparisons were performed using the Mann–Whitney test. *P < 0.05; **P < 0.01; ns, not significant. TCGA-PRAD, the Cancer Genome Atlas prostate adenocarcinoma; MSKCC, the Memorial Sloan Kettering Cancer Center; DKFZ, the German Cancer Research Center.

In the TCGA-PRAD cohort, GREM1 expression was significantly higher in tumors with Gleason score ≥8 than in those with Gleason score <8 (P = 0.0004) and in tumors with pathologic stage T3–T4 compared with T2 tumors (P < 0.05; **Figure 2**). The association with pathologic T stage was reproduced in both external cohorts, with higher GREM1 expression in T3-T4 tumors in MSKCC and DKFZ (both P < 0.01; **Figure 2**). Higher GREM1 expression in tumors with Gleason score ≥8 was also observed in DKFZ (P < 0.05), but not in MSKCC, where only 15 cases had a Gleason score ≥8. Overall, the association between GREM1 expression and pathologic T stage was consistent across all three cohorts, while the association with Gleason score was observed in two of the three cohorts.

### 3.3 Associations between GREM1 expression and clinical outcomes vary across independent cohorts

**Figure 3:**
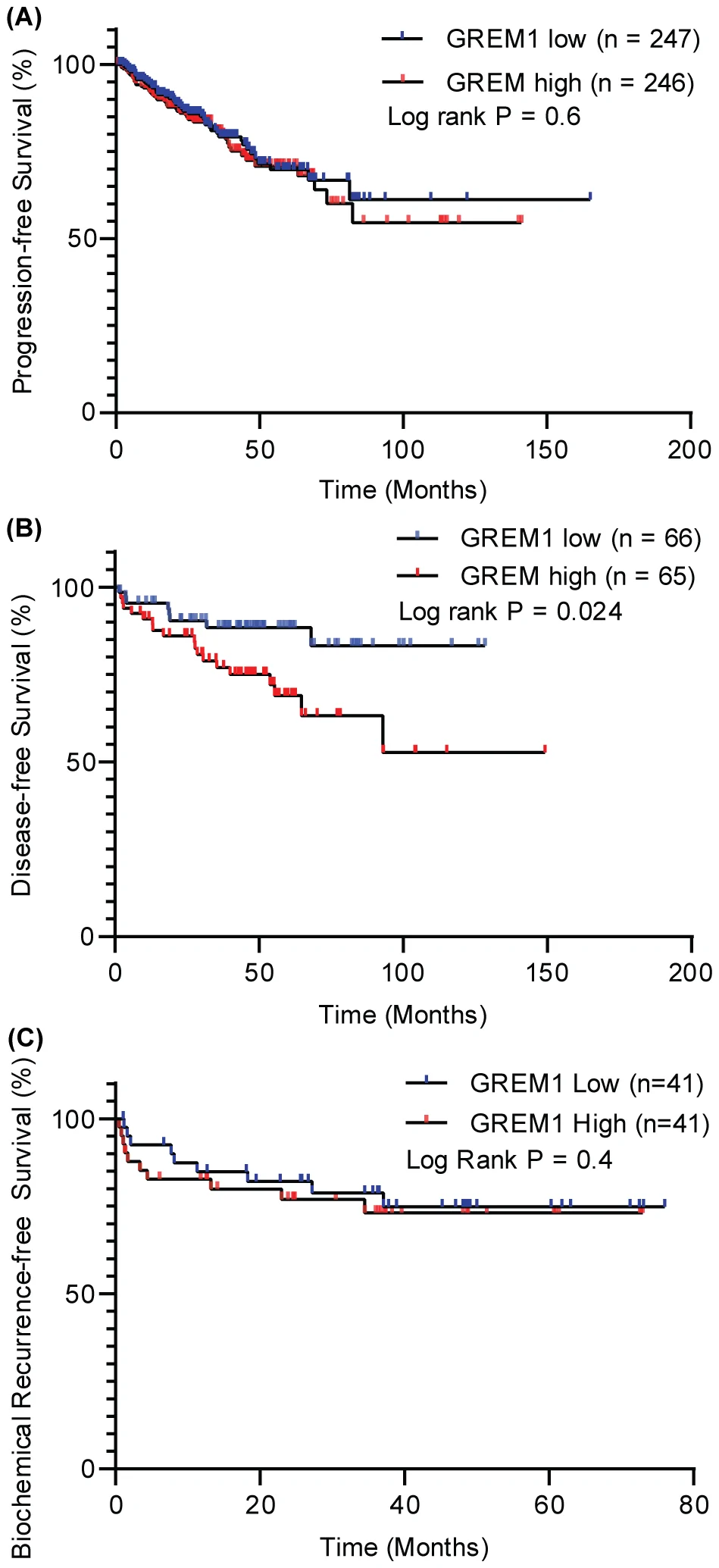
Kaplan-Meier survival analysis of GREM1 expression across three independent prostate cancer cohorts. **(A)** Progression-free survival (PFS) in the TCGA-PRAD cohort (PanCancer Atlas), comparing the GREM1-high (n = 246) and GREM1-low (n = 247) groups. **(B)** Disease-free survival (DFS) in the MSKCC cohort, comparing the GREM1-high (n = 65) and GREM1-low (n = 66) groups. **(C)** Biochemical recurrence-free survival in the DKFZ cohort, comparing the GREM1-high (n = 41) and GREM1-low (n = 41) groups. Gene expression and clinical data were obtained from cBioPortal. Patients in each cohort were divided into GREM1-high and GREM1-low groups using the cohort-specific median expression value. Survival analyses were performed using the Log-rank (Mantel–Cox) test. Tick marks indicate censored cases. TCGA-PRAD, the Cancer Genome Atlas prostate adenocarcinoma; MSKCC, the Memorial Sloan Kettering Cancer Center; DKFZ, the German Cancer Research Center.

To assess the association between GREM1 expression and clinical outcome, Kaplan–Meier survival analysis was performed in three independent cohorts after dividing patients into GREM1-high and GREM1-low groups using the cohort-specific median expression value. In the MSKCC cohort, the GREM1-high group had significantly shorter disease-free survival than the GREM1-low group (Log-rank P = 0.024). In contrast, no significant difference was observed in progression-free survival in the TCGA-PRAD cohort (Log-rank P = 0.60) or in biochemical recurrence-free survival in the DKFZ cohort (Log-rank P = 0.40) (**Figure 3**).

### 3.4 GREM1 expression does not demonstrate independent prognostic value after clinicopathologic adjustment

To evaluate whether GREM1 expression was independently associated with clinical outcomes, univariable and multivariable Cox regression analyses were performed in the three cohorts, with GREM1 analyzed as a continuous variable. In the MSKCC cohort, GREM1 was significantly associated with disease-free survival in the univariable analysis (HR = 2.34, 95% CI 1.09–4.72, *P* = 0.024). However, this association was not statistically significant after adjustment for Gleason score and pathologic T stage (HR = 1.40, 95% CI 0.68–2.72, *P* = 0.335). No significant association was observed in either univariable or multivariable analyses in the TCGA-PRAD or DKFZ cohorts (**Table 1**). Similar results were obtained when GREM1 was adjusted for pathologic T stage or Gleason score separately (**Supplementary Table 2**). Adding GREM1 to a model containing pathologic T stage and Gleason score did not improve model fit in any cohort, based on AIC (**Supplementary Table 2**). Overall, these findings suggest that GREM1 expression did not demonstrate independent prognostic value after clinicopathologic adjustment in any of the three cohorts.

**Table 1:** Univariable and multivariable Cox regression of GREM1 expression across three independent cohorts.

Cohort (n)	Model	GREM1 HR (95% CI)	P
MSKCC (n=131)	Univariate	2.338 (1.085–4.716)	0.0238
	GREM1+ T-stage + Gleason	1.403 (0.682–2.718)	0.3348
TCGA-PRAD (n=493/486)	Univariate	1.065 (0.954–1.197)	0.273
	GREM1+ T-stage + Gleason	0.994 (0.895–1.110)	0.9123
DKFZ (n=82)	Univariate	1.184 (0.741–1.876)	0.4767
	GREM1+ T-stage + Gleason	0.836 (0.501–1.333)	0.4695

GREM1 expression was analyzed as a continuous log2;-transformed variable. Models were adjusted for pathologic T stage (T2 vs. T3–T4) and Gleason score (<8 vs. ≥8). Survival endpoints were disease-free survival (MSKCC), progression-free survival (TCGA-PRAD), and biochemical recurrence-free survival (DKFZ).

TCGA-PRAD, the Cancer Genome Atlas prostate adenocarcinoma; MSKCC, the Memorial Sloan Kettering Cancer Center; DKFZ, the German Cancer Research Center.

### 3.5 GREM1 expression correlates with extracellular matrix and AR-related genes

To evaluate the transcriptional context associated with GREM1, Spearman correlation analyses were performed in the TCGA-PRAD, MSKCC, and DKFZ cohorts, followed by false discovery rate correction. FAP showed the most consistent positive correlation with GREM1 across all three cohorts (TCGA-PRAD: r = 0.523, q < 0.0001; MSKCC: r = 0.330, q = 0.0013; DKFZ: r = 0.426, q = 0.0013; **Table 2**). ACTA2 was positively correlated with GREM1 only in TCGA-PRAD after correction. Positive correlations with multiple TGF-β–related genes were observed in TCGA-PRAD, whereas only TGFBR1, TGFBR2, and SMAD2 remained significant in DKFZ, and none reached significance in MSKCC.

**Table 2:** Correlation of GREM1 with TGF-β signaling genes, fibroblast/extracellular matrix (ECM) markers, and canonical androgen receptor (AR)-related genes in three independent prostate cancer cohorts.

Category	Gene	TCGA			MSKCC			DKFZ			Pattern
		r	P	Adjusted q	r	P	Adjusted q	r	P	Adjusted q	
TGF-β signaling	TGFB1	0.274	<0.0001	<0.0001	0.049	0.576	0.6964	-0.027	0.7943	0.8442	Opposite direction in DKFZ; not significant in DKFZ/MSKCC
	TGFB2	0.214	<0.0001	<0.0001	0.116	0.1881	0.4891	0.046	0.6571	0.7766	Consistent direction; not significant in DKFZ/MSKCC
	TGFBR1	0.175	<0.0001	<0.0001	0.048	0.5888	0.6964	0.252	0.0137	0.0445	Consistent direction; significant in DKFZ, not MSKCC
	TGFBR2	0.215	<0.0001	<0.0001	0.058	0.5071	0.6964	0.229	0.0254	0.0472	Consistent direction; significant in DKFZ, not MSKCC
	SMAD2	0.187	<0.0001	<0.0001	0.023	0.7976	0.7976	0.341	0.0007	0.0046	Consistent direction; significant in DKFZ, not MSKCC
	SMAD3	0.197	<0.0001	<0.0001	0.024	0.789	0.7976	0.02	0.8442	0.8442	Consistent direction; not significant in DKFZ/MSKCC
Fibroblast/ECM marker	FAP	0.523	<0.0001	<0.0001	0.33	0.0001	0.0013	0.426	<0.0001	0.0013	Consistent in all three cohorts, all significant
	ACTA2	0.189	<0.0001	<0.0001	0.122	0.1666	0.4891	-0.17	0.1003	0.1449	Opposite direction in DKFZ; not significant in DKFZ/MSKCC
AR-related gene	AR	0.07	0.0995	0.0995	-0.073	0.4055	0.6964	0.201	0.0508	0.0826	Opposite direction in MSKCC; not significant in any cohort
	KLK3 (PSA)	-0.38	<0.0001	<0.0001	-0.142	0.1054	0.4593	-0.276	0.0068	0.0295	Consistent direction; significant in DKFZ, not MSKCC
	KLK2	-0.38	<0.0001	<0.0001	-0.095	0.2815	0.6099	-0.237	0.0208	0.0472	Consistent direction; significant in DKFZ, not MSKCC
	TMPRSS2	-0.31	<0.0001	<0.0001	-0.142	0.106	0.4593	-0.233	0.023	0.0472	Consistent direction; significant in DKFZ, not MSKCC
	NKX3-1	-0.09	0.048	0.052	-0.048	0.5893	0.6964	0.119	0.2503	0.3254	Opposite direction in DKFZ; not significant in DKFZ/MSKCC; not significant after FDR correction in TCGA

Spearman correlation coefficients (r), P values, and adjusted q values for the correlations between GREM1 and 13 genes in the TCGA-PRAD (Firehose Legacy, n = 493), MSKCC (n = 131), and DKFZ (n = 82) cohorts. P values were adjusted using the Benjamini–Hochberg false discovery rate method. An adjusted q value < 0.05 was considered statistically significant.

TCGA-PRAD, the Cancer Genome Atlas prostate adenocarcinoma; MSKCC, the Memorial Sloan Kettering Cancer Center; DKFZ, the German Cancer Research Center.

Among the AR-related genes, KLK3, KLK2, and TMPRSS2 were negatively correlated with GREM1 in all three cohorts. These correlations remained statistically significant after FDR correction in TCGA-PRAD and DKFZ but not in MSKCC. AR transcript levels were not significantly correlated with GREM1 in any cohort. The weak inverse correlation with NKX3–1 in TCGA-PRAD was no longer significant after FDR correction (q = 0.052). Overall, these findings identify FAP as the most reproducible transcriptional correlate of GREM1, while the associations with TGF-β-related and AR-related genes varied across cohorts.

### 3.6 Quantitative analysis demonstrates higher epithelial than stromal GREM1 expression

**Figure 4:**
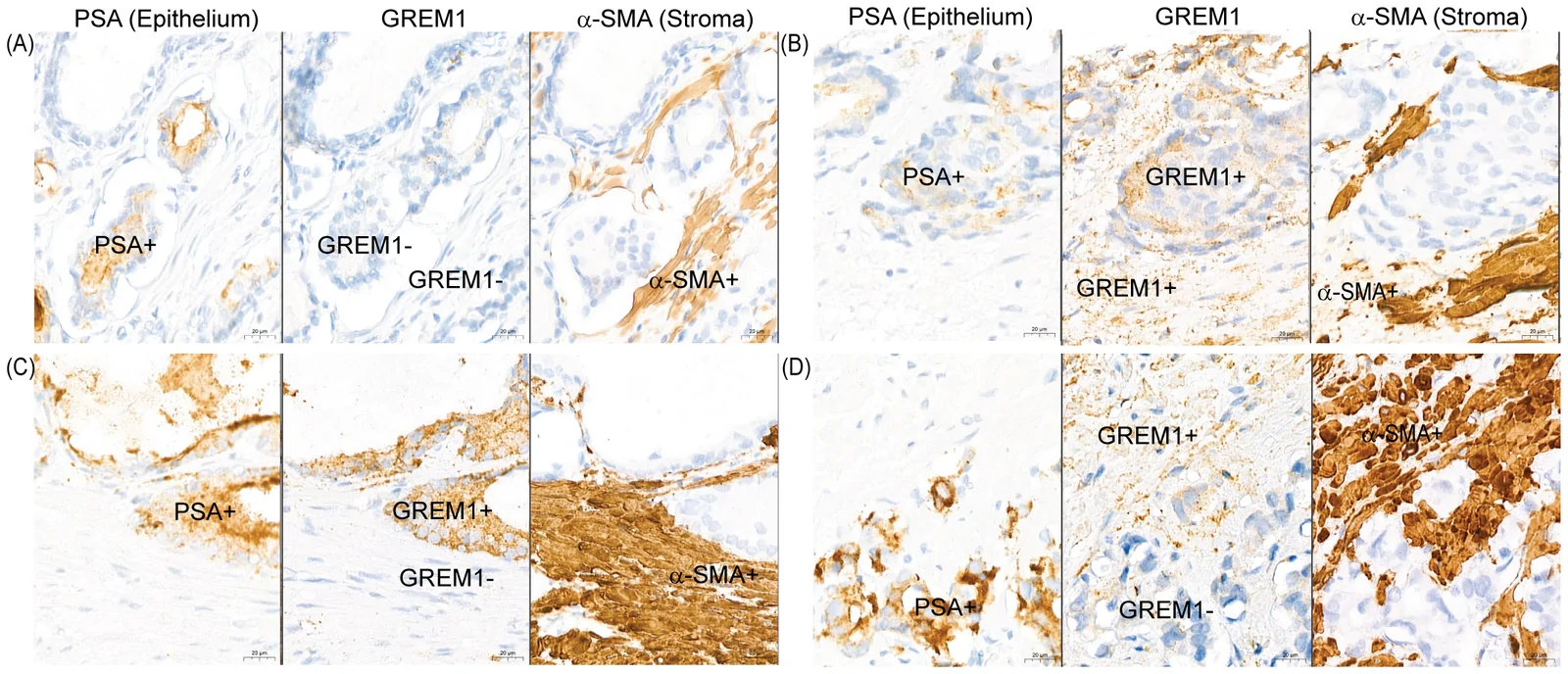
Immunohistochemical staining of GREM1 in human prostate tumor tissue microarrays (TMAs). In each set, PSA staining in the left panels and α-SMA staining in the right panels were used to identify epithelial and stromal compartments, respectively. GREM1 staining is shown in the middle panels. Representative tumor cores demonstrate **(A)** absence of GREM1 staining in both epithelial and stromal compartments, **(B)** GREM1 positivity in both epithelial and stromal compartments, **(C)** predominant epithelial GREM1 staining with minimal or absent stromal staining, and **(D)** predominant stromal GREM1 staining with little or no epithelial staining. Scale bars = 20 μm.

To characterize the compartment distribution of GREM1 protein expression, 87 evaluable prostate cancer TMA cores were examined by immunohistochemistry. Representative cores demonstrated heterogeneous staining patterns, including absent staining in both compartments (**Figure 4A**), staining in both epithelial and stromal regions (**Figure 4B**), epithelial-predominant staining (**Figure 4C**), and stromal-predominant staining (**Figure 4D**). PSA and α-SMA staining on adjacent sections supported the identification of epithelial and stromal compartments, respectively.

**Figure 5:**
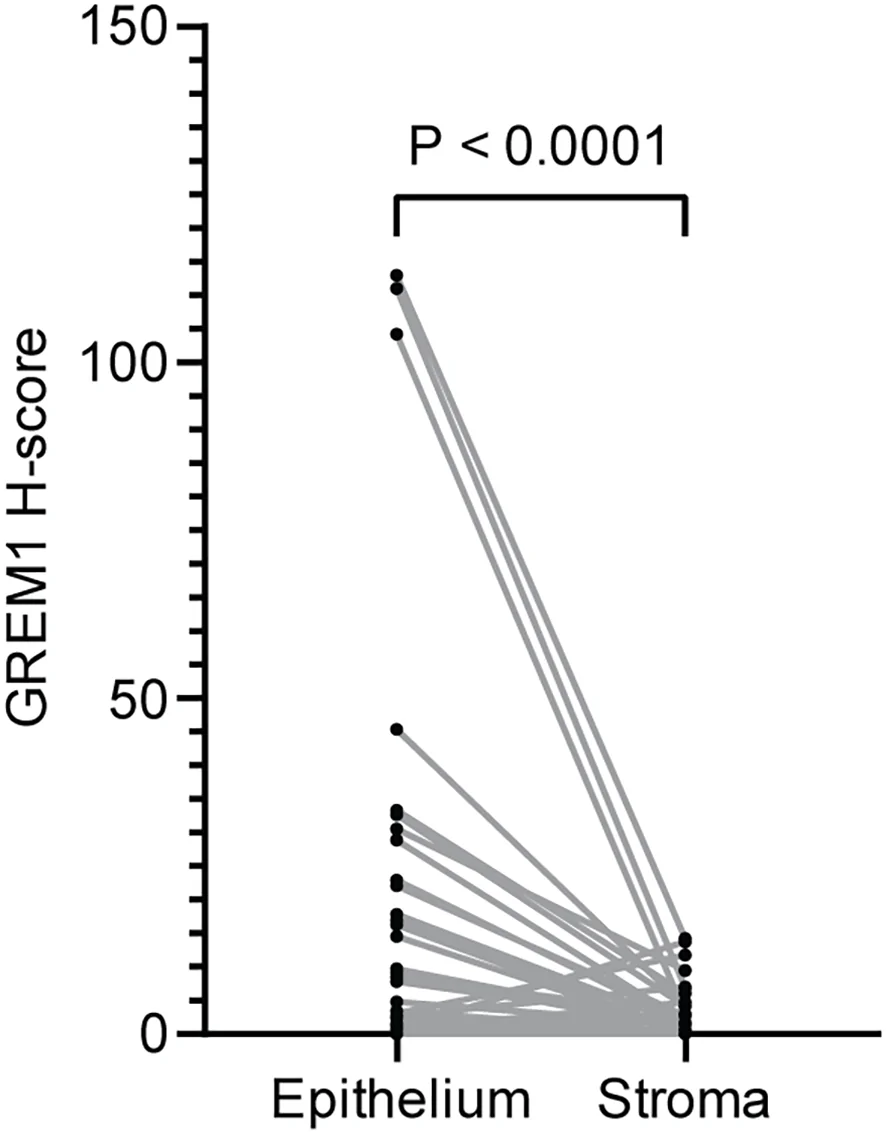
Quantitative comparison of epithelial and stromal GREM1 expression in prostate cancer tissue microarrays (TMAs).. GREM1 immunostaining was quantified separately in the epithelial and stromal compartments using H-scores derived from digital image analysis in QuPath. Each pair of connected points represents matched epithelial and stromal H-scores from the same patient (n = 43). When duplicate tissue microarray cores were available, the mean H-score across the cores was used for analysis. Epithelial GREM1 H-scores were significantly higher than the corresponding stromal H-scores (two-tailed Wilcoxon matched-pairs signed-rank test, P < 0.0001).

After averaging multiple evaluable cores from the same patient, 43 patients had paired epithelial and stromal H-score measurements. The median epithelial H-score was 3.10 (IQR, 0.76–17.83), compared with 1.41 (IQR, 0.33–3.00) in the stromal compartment. Epithelial H-scores were significantly higher than paired stromal H-scores (Wilcoxon matched-pairs signed-rank test, P < 0.0001; **Figure 5**). The median within-patient difference, calculated as stromal minus epithelial H-score, was −2.81. These findings quantitatively demonstrate higher epithelial than stromal GREM1 expression, although substantial intertumoral heterogeneity was observed.

Neither epithelial nor stromal GREM1 H-scores differed significantly between tumors with Gleason score <8 and those with Gleason score ≥8, or between pathologic stage pT2 and pT3-pT4 tumors (all P > 0.05).

## 4 Discussion

The present study integrated public transcriptomic datasets with quantitative immunohistochemistry of epithelial and stromal compartments to characterize the clinicopathologic features, clinical outcomes and molecular correlates of GREM1 expression in primary prostate cancer. Across the TCGA-PRAD, MSKCC, and DKFZ cohorts, higher GREM1 mRNA expression in primary prostate cancer was consistently associated with advanced pathologic T stage, whereas its association with Gleason score was observed in TCGA-PRAD and DKFZ but not in MSKCC. FAP was the most reproducible transcriptional correlate of GREM1 across cohorts, whereas selected AR-related transcripts showed inverse correlations with GREM1 across all three cohorts, although their statistical significance varied. Higher GREM1 expression was associated with shorter disease-free survival in MSKCC, but this association was not significant after clinicopathologic adjustment, and no significant outcome associations were observed in TCGA-PRAD or DKFZ. At the protein level, quantitative analysis demonstrated higher epithelial than stromal GREM1 H-scores. However, neither compartment-specific H-score was associated with Gleason score or pathologic T stage. Together, these findings associate GREM1 with adverse pathologic features, a fibroblast-associated transcriptional context, and lower expression of selected AR-related genes. Quantitative immunohistochemistry demonstrated heterogeneous GREM1 expression in both epithelial and stromal compartments, with higher epithelial H-score on average. Nevertheless, the present data does not establish a causal role or independent prognostic value for GREM1. In the TCGA-PRAD PanCancer Atlas cohort, GREM1 expression was higher in prostate cancer and bladder cancer compared with other genitourinary cancer types. Within primary prostate cancer, higher GREM1 expression was associated with advanced pathologic T stage across all three cohorts and with Gleason score ≥8 in TCGA-PRAD and DKFZ, supporting an association with adverse clinicopathologic features. These findings are consistent with the study by Cheng et al., which showed that GREM1 expression is further increased in CRPC compared with primary disease and is enriched in AR-indifferent states such as double-negative prostate cancer (DNPC) (10). Our study extends these findings by showing that the association between GREM1 expression and adverse pathologic features is detectable in primary prostate adenocarcinoma and reproducible across independent cohorts, although these observational findings do not provide direct evidence indicating that GREM1 drives disease progression. Previous studies have also reported elevated GREM1 expression in multiple malignancies and have linked it to tumor progression and microenvironmental remodeling (6, 16–18), further supporting the biologic relevance of GREM1 in aggressive cancer states. Although Cheng et al. reported an association between higher GREM1 expression and adverse clinical outcomes (10), its prognostic relevance was less consistent across the cohorts examined in our study. Higher GREM1 expression was associated with shorter disease-free survival in MSKCC, whereas no significant association was observed with progression-free survival in TCGA-PRAD or biochemical recurrence-free survival in DKFZ. Furthermore, the association in MSKCC was attenuated after adjustment for Gleason score and pathologic T stage, and GREM1 was not independently associated with clinical outcomes in any cohort. Differences in clinical endpoints, cohort size, event frequency, follow-up, and patient composition may have contributed to the variation across cohorts. Collectively, these findings suggest that GREM1 expression may reflect adverse pathologic or microenvironmental features but does not, by itself, provide prognostic information beyond established clinicopathologic factors in primary prostate cancer.

Our study also places GREM1 within a fibroblast-associated and stromal-remodeling transcriptional context. FAP was the most reproducible correlate of GREM1, showing a positive association across all three cohorts. Positive correlations with several TGF-β signaling components, including TGFB1, TGFB2, TGFBR1, TGFBR2, SMAD2, and SMAD3, and ACTA2 were observed predominantly in TCGA-PRAD, whereas only a subset of TGF-β-related genes remained significantly correlated with GREM1 in DKFZ, and none reached significance in MSKCC after FDR correction. This variation across cohorts argues against interpreting GREM1 as a uniform marker of TGF-β pathway activation, while the consistent association with FAP supports its relationship with a fibroblast-associated transcriptional context. These findings are consistent with prior studies showing that GREM1 is enriched in cancer-associated fibroblasts (CAFs), including myofibroblastic subtypes, and can promote tumor progression through paracrine mechanisms. In particular, GREM1 has been implicated in the regulation of cancer stem cell properties and in facilitating epithelial–mesenchymal transition (EMT) (16, 19, 20). Such CAF-enriched, tumor-reactive stroma has been associated with adverse clinical outcomes and biochemical recurrence in prostate cancer (21, 22). However, because bulk transcriptomic correlations cannot distinguish tumor-cell expression from stromal-cell expression or differences in tissue composition, the present findings support an association with stromal remodeling programs but do not establish cell-specific functional interactions. In parallel, GREM1 showed consistently negative correlations with selected AR-related genes, particularly KLK3, KLK2 and TMPRSS2, across all three cohorts, although these associations remained significant after FDR correction only in TCGA-PRAD and DKFZ. AR transcript levels themselves were not significantly correlated with GREM1 expression in any cohort, and the weak inverse correlation with NKX3–1 in TCGA-PRAD did not remain significant after FDR correction. Thus, these findings associate higher GREM1 expression with lower expression of selected AR-related transcripts, which should not be interpreted as evidence of globally reduced AR activity or direct suppression of AR signaling by GREM1. This aligns with recent studies by Cheng et al. (10, 23), which identified GREM1 as an alternative ligand for FGFR1 capable of sustaining tumor growth and MAPK signaling under conditions of AR suppression. However, the present transcriptomic analyses in primary tumors do not directly test this mechanism. Taken together, GREM1 expression may be linked to tumor states characterized by lower expression of selected AR-related genes, although direct mechanistic relationships remain to be established. Interestingly, our tissue-level analyses revealed a spatial pattern that differs from the conventional view of GREM1 as a predominantly stromal factor (5–8). Quantitative immunohistochemical analysis of human prostate cancer TMAs demonstrated higher epithelial than stromal GREM1 H-scores on average, although expression was heterogeneous and detectable in both compartments. This finding is supported by previous studies showing that GREM1 protein can be detected within tumor cells across multiple cancer types. In an analysis of 208 tumor samples, GREM1 was identified in tumor cells in the majority of cases, including glioblastoma, hepatocellular carcinoma, invasive lobular breast carcinoma, and diffuse B-cell lymphoma (9). In colorectal cancer, aberrant epithelial GREM1 expression has been shown to disrupt normal morphogen gradients and promote tumor initiation through ectopic crypt formation (24). Another report about epithelial GREM1 expression in colorectal cancer showed that GREM1 was mainly detected in stromal fibroblasts in early-stage tumors, whereas GREM1-positive epithelial tumor cells were identified almost exclusively in stage IV disease. In that study, epithelial GREM1–ACVR1C signaling was associated with EMT, metastasis, and poor survival (25). In pancreatic ductal adenocarcinoma, GREM1 expression has been detected in both stromal and tumor epithelial compartments, with evidence suggesting a transition from predominantly stromal localization in precursor lesions to combined stromal and epithelial expression in invasive tumors (5). A recent review has similarly highlighted the context-dependent stromal and tumor-cell roles of GREM1 in cancer and fibrosis (26). The review describes interactions among GREM1, fibroblast activation, BMP/TGF-β signaling, and extracellular matrix remodeling, providing a biological framework for the consistent positive correlation between GREM1 and FAP observed across our three cohorts. Although these findings suggest that GREM1 may participate in tumor-stromal signaling and represent a potential therapeutic target, our observational analyses do not establish causality or therapeutic efficacy. Taken together, these studies indicate that epithelial GREM1 localization is not unique to prostate cancer and may occur across multiple tumor types during disease progression, although its biological and clinical significance in primary prostate cancer remains to be established.

Several limitations should be acknowledged. First, this retrospective observational study integrated public bulk transcriptomic datasets with TMA-based immunohistochemistry. Therefore, the observed associations should not be interpreted as causal, and transcriptomic GREM1 expression and gene-level correlations cannot be definitively attributed to epithelial or stromal cells. Second, the transcriptomic cohorts differed in sample size, clinical characteristics, follow-up, and outcome definitions, limiting direct comparisons. Treatment exposure and response data were not sufficiently complete or consistently annotated to permit reliable analysis, and residual confounding may remain despite adjustment for Gleason score and pathologic T stage. Third, transcriptomic and protein-level analyses were conducted in independent cohorts, precluding patient-level comparison of GREM1 mRNA and protein expression. Fourth, the TMA cohort was relatively small (43 patients with paired measurements), lacked clinical outcome data, and included limited cores that may not fully capture intratumoral heterogeneity. Although epithelial and stromal regions were separately annotated and supported by adjacent PSA and α-SMA staining, we plan to use multiplex immunofluorescence or spatially resolved methods in future studies to confirm the cellular origin of GREM1 expression. Fifth, potential cross-reactivity of the custom GREM1 antibody with other BMP antagonists, including GREM2, was not evaluated. Finally, the absence of functional studies precludes conclusions regarding the direct role of GREM1 in prostate cancer progression. Larger clinically annotated cohorts, orthogonal protein-level validation, and mechanistic studies are needed.

## 5 Conclusion

In conclusion, GREM1 mRNA expression in primary prostate cancer was associated with adverse pathologic features, a fibroblast-associated transcriptional context, and lower expression of selected AR-related genes, but did not demonstrate independent prognostic value. At the protein level, GREM1 expression was heterogeneous in both epithelial and stromal compartments, with higher epithelial H-score on average. These findings support further investigation of compartment-specific GREM1 expression and its biological relevance in prostate cancer.

## Data Availability

The raw data supporting the conclusions of this article will be made available by the authors, without undue reservation.
